# Flow-compensated diffusion encoding in MRI for improved liver metastasis detection

**DOI:** 10.1371/journal.pone.0268843

**Published:** 2022-05-26

**Authors:** Frederik B. Laun, Tobit Führes, Hannes Seuss, Astrid Müller, Sebastian Bickelhaupt, Alto Stemmer, Thomas Benkert, Michael Uder, Marc Saake

**Affiliations:** 1 Institute of Radiology, University Hospital Erlangen, Friedrich-Alexander-Universität Erlangen-Nürnberg, Erlangen, Germany; 2 Department of Radiology, Klinikum Forchheim—Fränkische Schweiz gGmbH, Forchheim, Germany; 3 Siemens Healthcare GmbH, Erlangen, Germany; Medical University of Vienna, AUSTRIA

## Abstract

Magnetic resonance (MR) diffusion-weighted imaging (DWI) is often used to detect focal liver lesions (FLLs), though DWI image quality can be limited in the left liver lobe owing to the pulsatile motion of the nearby heart. Flow-compensated (FloCo) diffusion encoding has been shown to reduce this pulsation artifact. The purpose of this prospective study was to intra-individually compare DWI of the liver acquired with conventional monopolar and FloCo diffusion encoding for assessing metastatic FLLs in non-cirrhotic patients. Forty patients with known or suspected multiple metastatic FLLs were included and measured at 1.5 T field strength with a conventional (monopolar) and a FloCo diffusion encoding EPI sequence (single refocused; b-values, 50 and 800 s/mm^2^). Two board-certified radiologists analyzed the DWI images independently. They issued Likert-scale ratings (1 = worst, 5 = best) for pulsation artifact severity and counted the difference of lesions visible at b = 800 s/mm² separately for small and large FLLs (i.e., < 1 cm or > 1 cm) and separately for left and right liver lobe. Differences between the two diffusion encodings were assessed with the Wilcoxon signed-rank test. Both readers found a reduction in pulsation artifact in the liver with FloCo encoding (p < 0.001 for both liver lobes). More small lesions were detected with FloCo diffusion encoding in both liver lobes (left lobe: six and seven additional lesions by readers 1 and 2, respectively; right lobe: five and seven additional lesions for readers 1 and 2, respectively). Both readers found one additional large lesion in the left liver lobe. Thus, flow-compensated diffusion encoding appears more effective than monopolar diffusion encoding for the detection of liver metastases.

## Introduction

Diffusion-weighted imaging (DWI) has become an indispensable technique for the detection of focal liver lesions (FLLs) [1]. The presence or absence of FLL is a key parameter for choosing the subsequent treatment path in many tumor diseases, e.g. neuroendocrine tumors [2]. In liver DWI, as in virtually all other fields of clinical DWI, the diffusion encoding is based on variations of the pulsed gradient approach introduced by Stejskal and Tanner [3], which is for good reason; no other gradient time profile can match its b-value efficiency [4, 5], at least if one compares versions optimized in this regard [6–9]. As a variation of this approach, twice-refocused diffusion encoding has found widespread application as a means for minimizing eddy current artifacts [10]. However, to our knowledge, no additional diffusion encodings have been widely applied in clinical MRI.

Nonetheless, a plethora of available diffusion encodings exist that exhibit fascinating properties, including oscillating gradients [11, 12], double diffusion encoding [13], and intensity-modulated two-gradient-pulse encoding [14], which all enable visualization of a range of microstructural tissue features, such as average cell surface-to-volume ratio [11] or even cell shape distribution (at least in certain limits) [14]. While extensively explored in a research context, it is presumably the high complexity of these advanced diffusion encodings, the need for extended scan time, and the rigorous technical demands that have so far prevented their transition into the broader clinical routine.

One specific diffusion encoding may stand out in this respect: flow-compensated (FloCo) diffusion encoding [15–20]. It suppresses the effect of signal decays induced by ballistic motion [15–18, 21] at the price of an increased minimally achievable echo time (TE) and a longer repetition time (TR). Overcoming these signal decays is relevant in several parts of the human body, but potentially nowhere to the same extent as in the left liver lobe due to its proximity to the heart [22, 23].

FloCo diffusion encoding can reduce the pulsation artifact considerably, as has been shown in a series of healthy volunteer investigations [6, 7, 24–26], although to our knowledge, until now, only one dataset from a single patient with FLLs has been presented [25]. In these patient images, a lesion was visible in the left liver lobe only with FloCo diffusion encoding. We are not aware of further studies clinically evaluating FloCo diffusion encodings for the detection of metastatic FLLs.

We hypothesized that a reduction in pulsation artifacts should lead to a more effective FLL detection in patients and conducted a prospective study to compare FloCo diffusion encoding to conventional monopolar diffusion encoding for the detection of liver metastases in oncologic patients.

## Materials and methods

### Study population

Patients aged ≥ 18 years were recruited prospectively from January to August 2020. To maximize the number of measured FLLs while minimizing the number of total scans, only patients who were known to have multiple malignant FLLs, or whose medical records made it likely that multiple malignant FLLs were present, were asked to participate in the study (stage IV cancer patients). Further inclusion criteria were a high probability that the patient would tolerate the examination prolonged by the study sequences, and written informed consent. Exclusion criteria were the presence of active or ferromagnetic implants, claustrophobia, tattoos close to the eyes, and sedative medications. All participants had a scheduled clinical MR examination and were recruited during the clinical workflow. The study was approved by the local Institutional Ethics Committee (study number, 276_19 B).

### Magnetic resonance imaging

All measurements were performed on a clinical 1.5 T scanner (MAGNETOM Aera, XQ gradients with max. gradient strength 45 mT/m and max. slew rate 200 T/m/s, Siemens Healthcare, Erlangen, Germany) with an 18-channel anterior body coil in combination with a 32-channel spine array coil.

A vendor-provided prototype echo planar imaging (EPI) sequence was used featuring two diffusion encodings: Monopolar and FloCo. Monopolar is the vendor-specific name for the single-refocused diffusion encoding scheme. The vendor-provided sequence option ‘dynamic field correction’ was used to compensate for eddy current induced image distortions [10]. In the FloCo diffusion encoding used in this work, two pairs of monopolar gradients are placed symmetrically around the single 180° refocusing pulse of the spin-echo EPI sequence. Note that this placement strategy of the diffusion encoding gradients results in a nulled zeroth gradient moment during the 180° pulses and thus makes the use of crusher gradients necessary that are placed besides the 180° pulses even for b-values larger than zero. These crusher gradients are not flow-compensated (and neither is the EPI readout) so that the flow-compensation is not perfect, strictly speaking. In the particular sequence implementation, the crusher gradients are merged with the diffusion encoding gradients. The TE of both DWI sequences was matched, to match the contrast-to-noise ratio (CNR). Table 1 summarizes the diffusion sequence parameters used to collect data before contrast agent administration.

**Table 1:** MRI sequence parameters for FloCo and monopolar DWI sequences.

Sequence	DWI EPI
Repetition time (ms)	12,400
Echo time (ms)	70
Voxel size (mm³)	3.125 × 3.125 × 5 interpolated to 1.6 × 1.6 × 5
Field of view (read × phase; mm²)	400 × 325
Phase direction	anterior-posterior
Phase resolution	100%
Partial Fourier	6/8
Matrix	128 × 104
Slice distance	20%
Number of slices	39 (axial)
Parallel imaging	GRAPPA ×2, 24 reference lines
Bandwidth (Hz/pixel)	2,790
Echo spacing (ms)	0.49
b-values (s/mm^2^)	50, 800
Averages (b50, b800)	1, 4
Diffusion mode	3-scan trace
Diffusion scheme	Once monopolar, once FloCo
Acquisition time (min:s)	3:43
Trigger	free breathing
Surface coil intensity correction	yes, the ‘pre-scan normalize’ option was used
Fat saturation	SPAIR & gradient reversal

DWI = diffusion-weighted imaging, EPI = echo planar imaging, GRAPPA = GeneRalized Autocalibrating Partial Parallel Acquisition, FloCo = flow-compensated, SPAIR = Spectral Adiabatic Inversion Recover

Additionally, a standard clinical liver MRI protocol was performed for each patient, consisting of a T2-weighted HASTE sequence (repetition time [TR], 1000 ms; echo time [TE], 92 ms; slice thickness [ST], 5 mm); a fat-saturated T2-weighted TSE sequence (TR, 4848 ms; TE, 102 ms; ST, 5 mm); and fat-saturated T1-weighted GRE sequences (TR, 7.22 ms; TE, 2.39 ms; ST, 5 mm), one taken before and several after contrast agent administration (0.1 mmol/kg body weight; gadobutrol, Gadovist/Gadavist, Bayer Vital, Leverkusen, Germany). The contrast agent was administered after the diffusion sequences.

### Image analysis

After an initial quality check, the readers only assessed FLLs that they identified as metastases. Concomitant benign lesions (e.g., cysts, hemangiomas) were not scored. The readers used all available image data and the clinical radiology reports to differentiate the lesions.

#### Quantitative evaluation

The minimal and maximal lesion size was measured in the FloCo b = 800 s/mm^2^ (b800) images using the ruler tool according to the Response Evaluation Criteria in Solid Tumors (RECIST 1.1 [27]) criteria by a trained physicist (T.F.) under the supervision of a board-certified radiologist (S.B.). The diameters were measured only in the FloCo b800 images because the lesions were best visible there.

Under the supervision of two radiologists (M.S. and S.B.), a trained physicist (T.F., 2 years of experience in abdominal DWI) defined 3D segmentations with the Medical Imaging Interaction Toolkit (MITK, v2021.02, Heidelberg, Germany), encompassing the largest lesion in the left and right liver lobes for both diffusion encodings. The 3D volumes were defined with enough distance to the lesion borders to avoid partial-volume effects and used to calculate the apparent diffusion coefficient (ADC). As parallel imaging had been used, the standard deviation (SD) of the noise depended on the position and could not be assessed easily, e.g., using the SD of the signal in a region outside the body. Thus, following [28–31], the SD of the liver parenchyma in the specified ROI was used. It was estimated with an approximately 10 cm² 2D segmentation drawn in a representative slice in the right liver lobe, sparing vessels. The signal-to-noise ratio (SNR) was then calculated by dividing the average signal in the 3D lesion segmentation by the standard deviation in the 2D liver segmentation.

The ADC was calculated from the signal averaged over the 3D lesion segmentations for three combinations (FloCo = b50-FloCo and b800-FloCo; Monopolar = b50-Monopolar and b800-Monopolar; Mixed = b50-Monopolar and b800-FloCo).

#### Qualitative whole-liver evaluation

Two board-certified radiologists (M.S. and H.S., 13 and 9 years of experience in abdominal imaging, respectively) independently rated the FloCo and monopolar datasets using 5-point Likert scales (1 = worst, 5 = best). The readers were not blinded to the acquisition type, as the distinction between the two techniques was obvious from the image impression.

To evaluate the image quality, the readers rated the following features (c.f. [31] for a detailed description of the scores):

- Overall image quality, once for b50 and once for b800, for both liver lobes combined (1 = very poor, 2 = poor, 3 = acceptable, 4 = good, 5 = very good), following [32–34].
- Blood signal blackness, once for b50 and once for b800, for both liver lobes combined (1 = very poor, 2 = poor, 3 = acceptable, 4 = good, 5 = very good).
- Severity of the cardiac motion artifact, once for the left and once for the right liver lobe, only for b800 as the artifact becomes only prominent at higher b-values (1 = liver lobe not identifiable, 2 = black holes frequently visible, 3 = strong signal loss and sporadic small black holes, 4 = slight signal loss, but no black holes, 5 = no signal loss visible), following [25].

#### Qualitative liver lesion evaluation

Following [31], the readers rated lesion conspicuity (Likert scale from 1 to 5) for small lesions (< 1 cm) and large lesions (≥ 1 cm) in each liver lobe. Lesion size was estimated visually and confirmed with the viewer’s ruler tool, if necessary.

Lesion detection performance was evaluated separately for small and large lesions. As patients with known or expected presence of multiple FLLs were included in this study, the absolute number of detected lesions was not recorded. Instead, the readers counted the number of lesions in the FloCo images and the monopolar images, and the difference in the number of lesions, expressed as *Δ*_lesions_, was calculated by subtracting the number of monopolar lesions from the number of FloCo lesions. A positive value indicated that there were more lesions found with FloCo than with monopolar encoding, while a negative value indicated that more lesions were found with monopolar than with FloCo encoding. To simplify the evaluation, Δ_lesions_ ≥ 3 was set to Δ_lesions_ = 3 and Δ_lesions_ ≤ -3 was set to Δ_lesions_ = -3.

### Statistical analysis

Statistical analysis was performed with MATLAB Release 2017b (The MathWorks, Inc., Natick, MA, USA). Significant differences in the absolute qualitative Likert scores between FloCo and monopolar diffusion encoding were tested using the non-parametric Wilcoxon signed-rank test. To assess the differences in SNR and ADC values between FloCo and monopolar diffusion encoding, the Shapiro–Wilk test was performed to test for normality. Subsequently, either a one-way ANOVA or a parametric test was performed (Wilcoxon signed-rank test or Kruskal–Wallis test). Additionally, post hoc tests were performed using Tukey’s honestly significant difference procedure. The inter-reader agreement was assessed by computing Cohen’s kappa (κ). A p-value < 0.05 was considered significant. The SNR, ADC, and smallest and largest visible lesions were described with descriptive statistics.

## Results

### Patients

**Fig 1:**
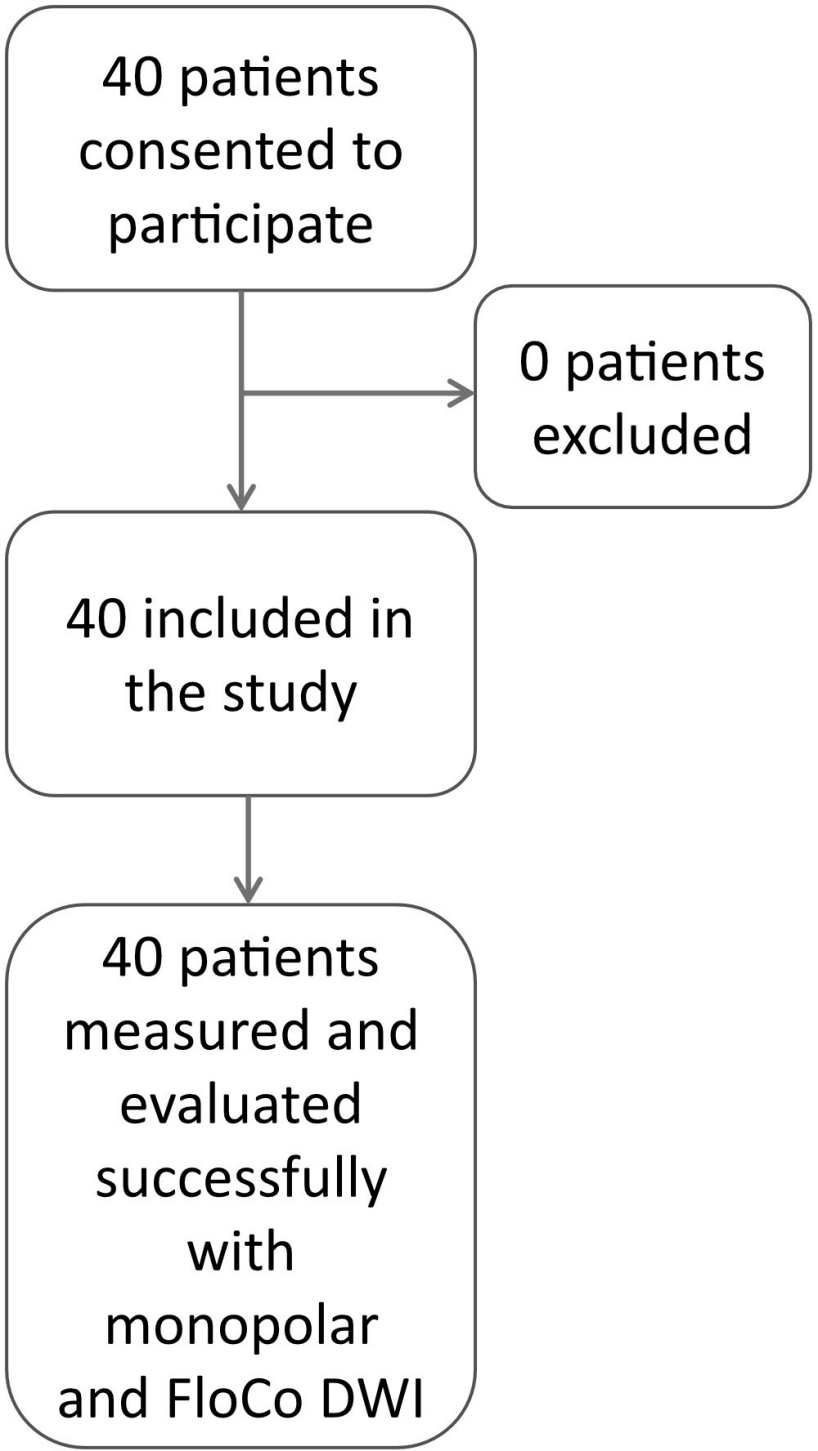
Participant flow diagram. Footnote: FloCo = Flow-compensated. DWI = diffusion-weighted imaging.

Forty consecutive patients were enrolled in this study (27 males, 13 females). The mean participant age was 60 ± 9 years (range: 34–74 years). All liver MRI scans were successfully and completely performed and passed the initial quality check. Quantitative and qualitative evaluations were performed for all participants. FLLs were present in all participants. Fig 1 displays the participant inclusion flow diagram and Table 2 summarizes further participant demographics and disease characteristics.

**Table 2:** Patient demographics and disease characteristics.

Disease	Number of patients
Neuroendocrine tumor	21/40 (52.5%)
Colorectal cancer	10/40 (25.0%)
Thyroid cancer	4/40 (10.0%)
Melanoma	2/40 (5.0%)
Mixed adeno-neuroendocrine carcinoma	1/40 (2.5%)
Pancreatic cancer	1/40 (2.5%)
Non-small cell lung cancer	1/40 (2.5%)
No evaluation of right liver lobe due to hemihepatectomy	3
No evaluation of left liver lobe due to hemihepatectomy	1
At least one lesion < 1 cm present in right liver lobe	34
At least one lesion > 1 cm present in right liver lobe	31
At least one lesion < 1 cm present in left liver lobe	32
At least one lesion > 1 cm present in left liver lobe	30

The disease type was confirmed histologically, though not for each assessed lesion.

### Representative images

**Fig 2:**
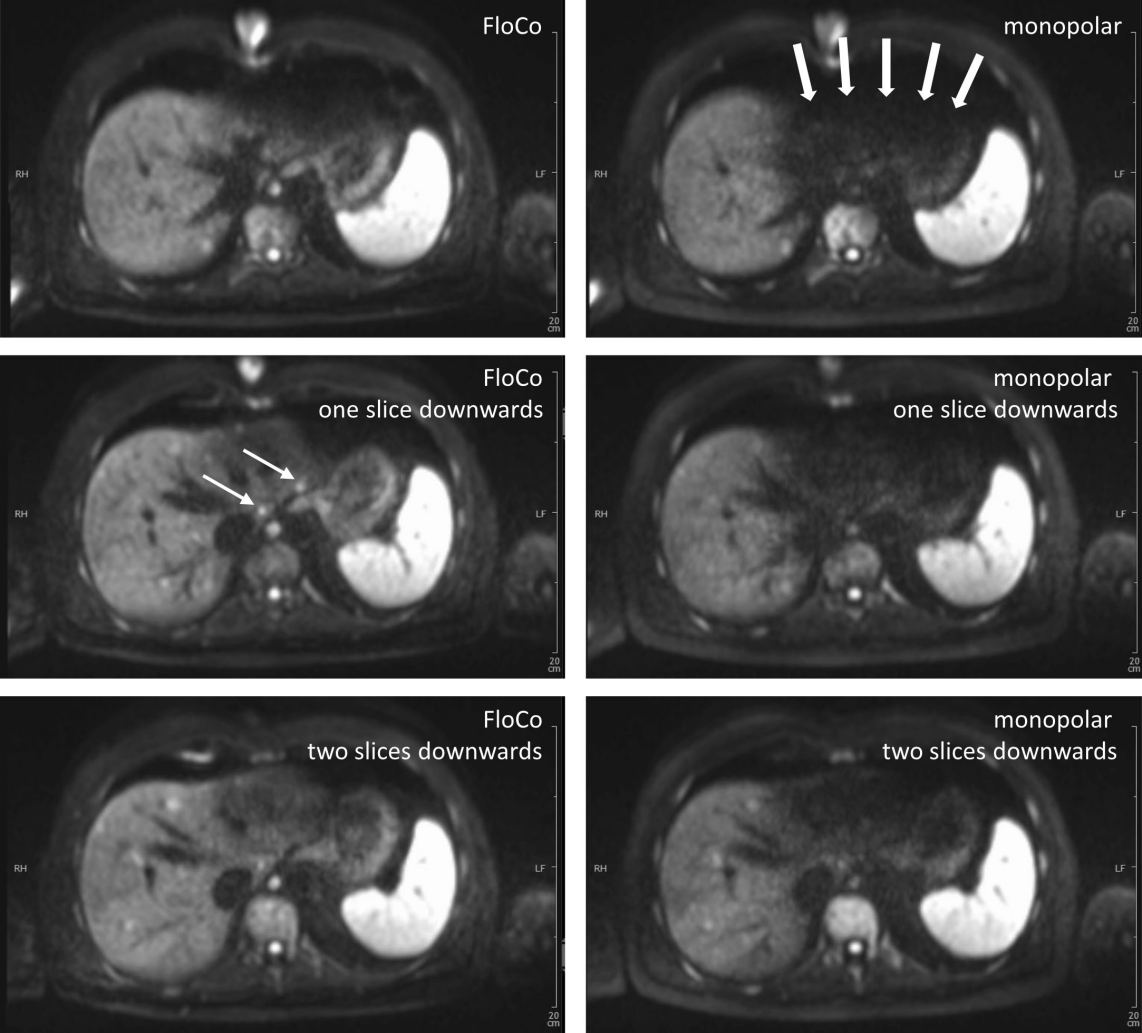
Diffusion-weighted b800 images of the liver in a 42-year-old patient with metastatic medullary thyroid carcinoma. Three adjacent slices, left-side flow-compensated (FloCo), right-side conventional monopolar diffusion encoding. There are considerable pulsation artifacts with monopolar diffusion encoding, which mask focal lesions in the left liver lobe (thick arrows). One of the arrow-marked small lesions was not reported by either of the two readers in the monopolar dataset. The other arrow-marked small lesion was not reported by one of the readers in the monopolar dataset.

**Fig 3:**
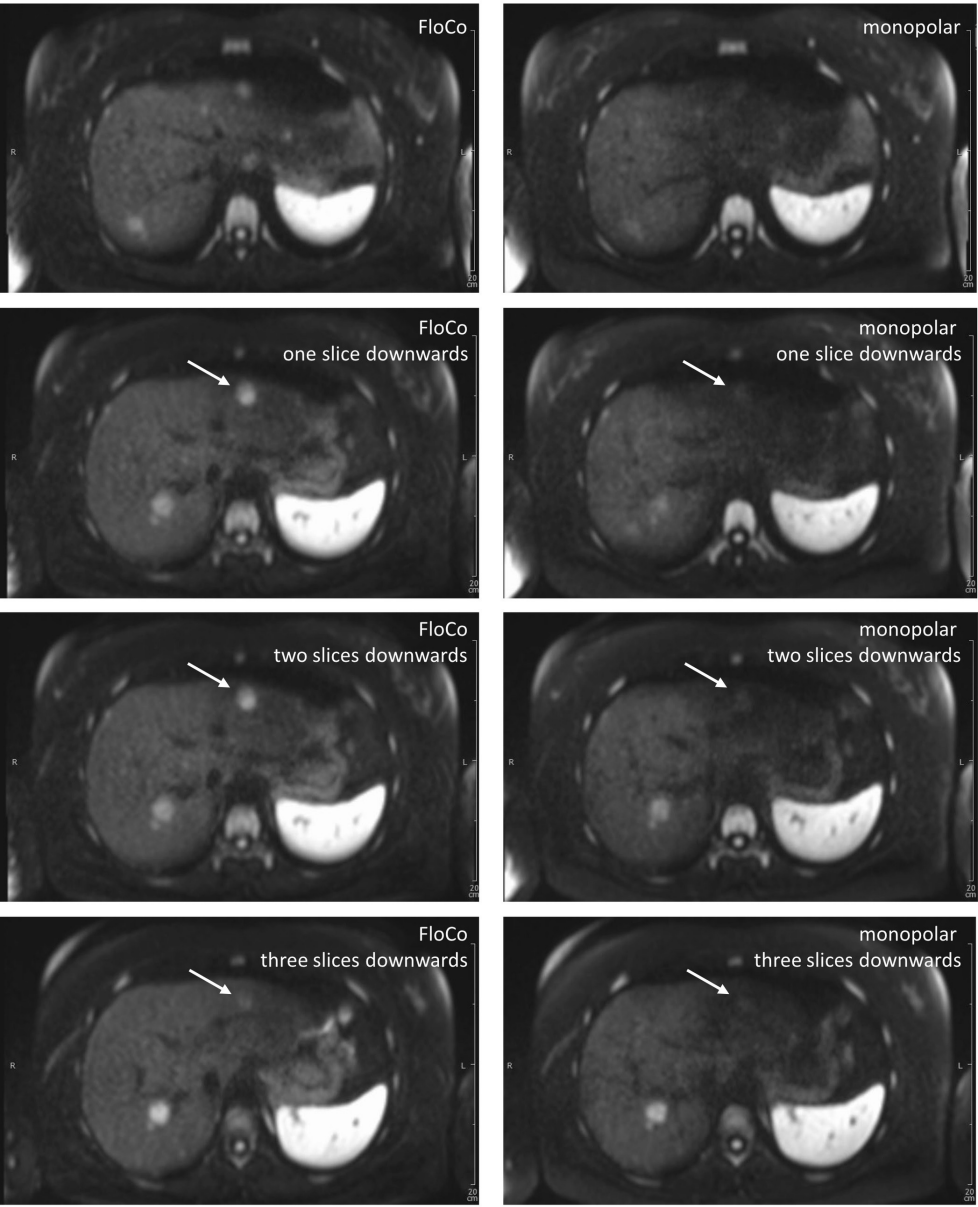
Diffusion-weighted b800 images of the liver in a 39-year-old patient with a metastatic neuroendocrine tumor of the jejunum. Four adjacent slices, left-side flow-compensated (FloCo), right-side monopolar diffusion encoding. The arrow-marked large lesion was detected by both readers but is much less visible in the monopolar dataset.

**Fig 4:**
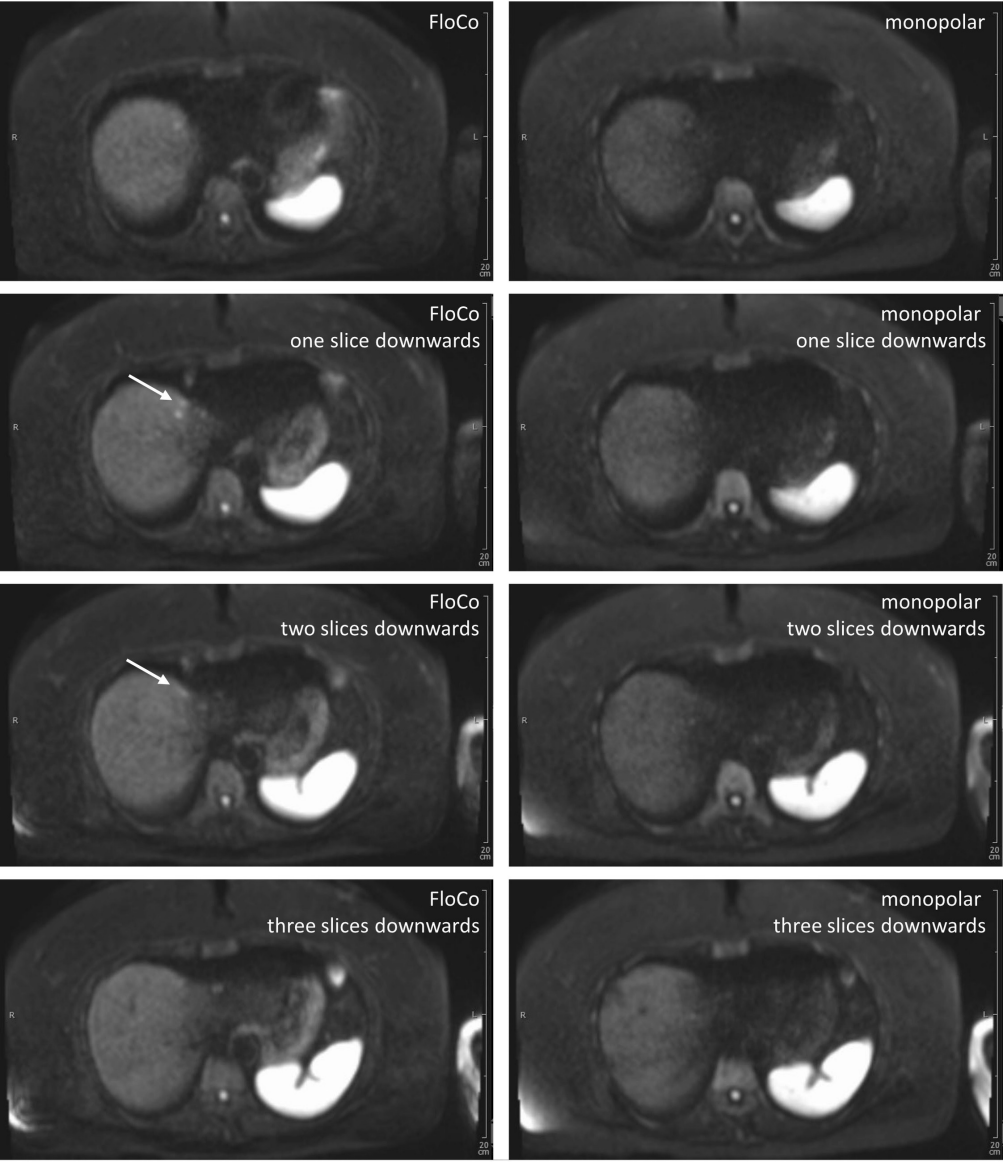
Diffusion-weighted b800 images of the liver in a 61-year-old patient with a metastatic neuroendocrine tumor of the ileum. Four adjacent slices, left-side flow-compensated (FloCo), right-side monopolar diffusion encoding. The arrow-marked small lesion was not reported by either of the two readers in the monopolar dataset. The adjacent lesion is barely visible in the monopolar dataset.

**Fig 5:**
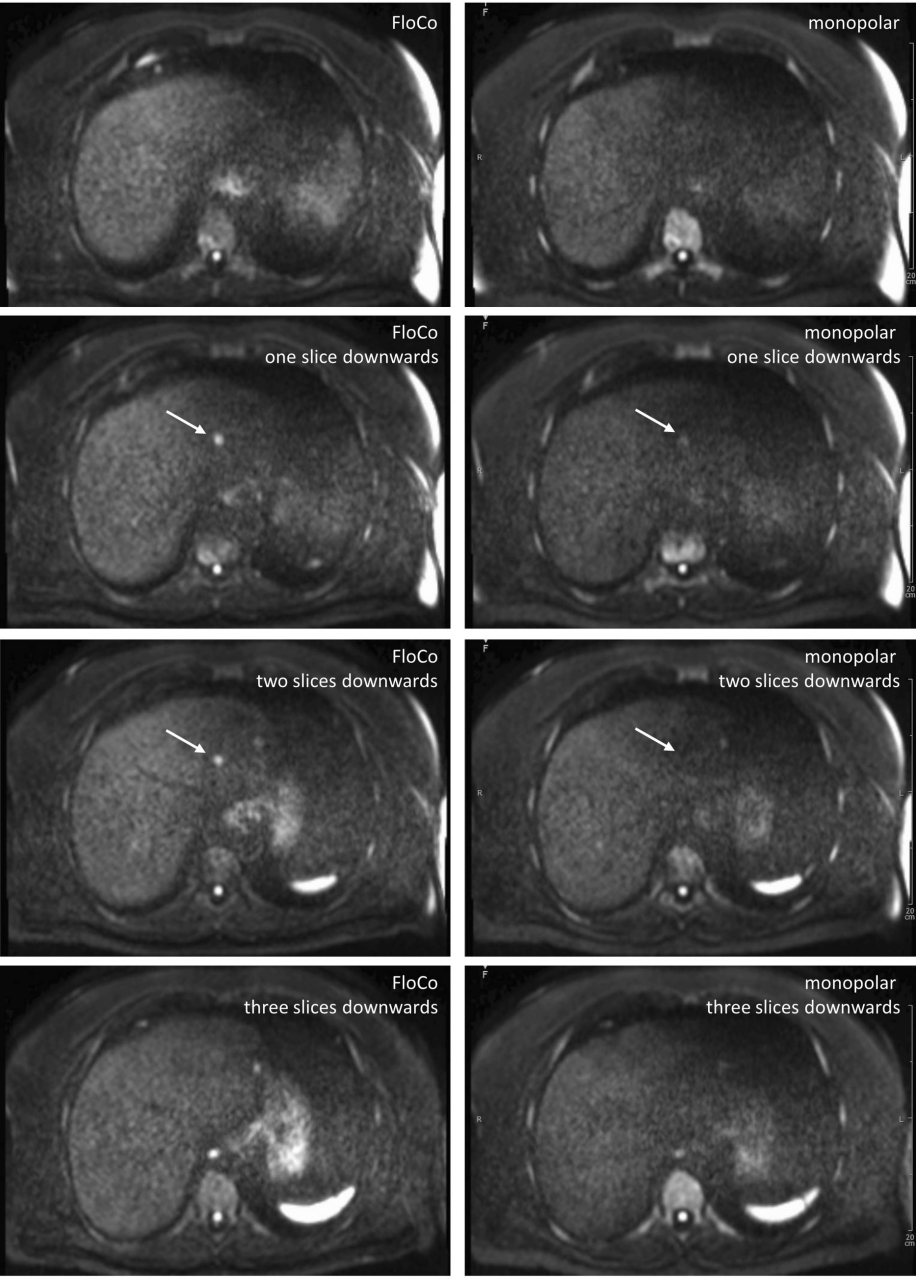
Diffusion-weighted b800 images of the liver in a 70-year-old patient with a metastatic neuroendocrine tumor of unknown primary. Four adjacent slices, left-side flow-compensated (FloCo), right-side monopolar diffusion encoding. The conspicuity was rated higher for the FloCo data by both readers (with a Likert score of 2).

Figs 2–5 illustrate representative cases. In all four examples, lesions were reported in the FloCo datasets and were missed or much less visible in the monopolar datasets.

### Quantitative image analysis

**Fig 6:**
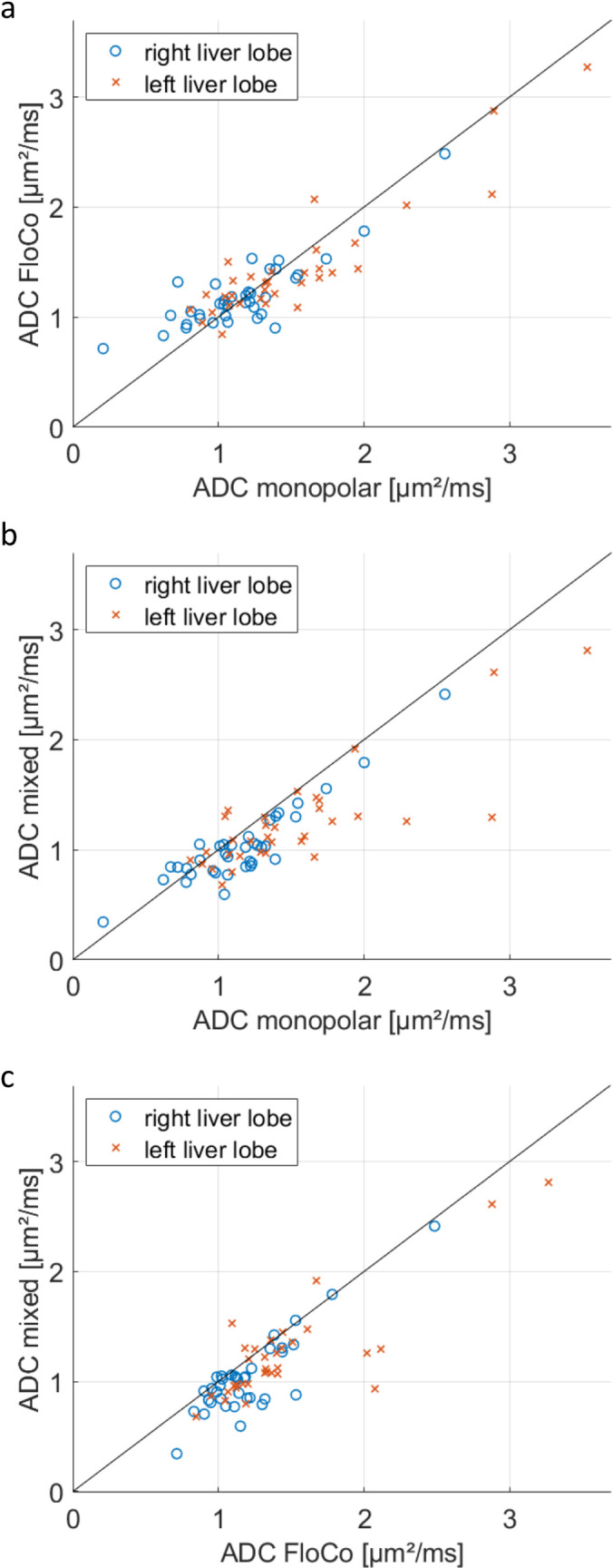
Individual subject ADC values for monopolar, FloCo, and mixed diffusion schemes.

Table 3 and Fig 6 summarize the quantitative and test statistics for the SNR in the largest lesion present in the right and left liver lobes measured with monopolar and FloCo acquisition at b800. Furthermore, the results for ADC and the diameter of the smallest and largest visible lesions in the left and right liver lobes are given. Small lesions were regularly present. A significant difference was found between the lesion SNRs measured with the two diffusion encodings for both liver lobes (one-way ANOVA for the left liver lobe; Wilcoxon signed-rank test for right liver lobe used because of failed Shapiro–Wilk test). The mean, median, minimum, quartile 1, quartile 3, and maximum SNR in the left liver lobe lesions were approximately 30% higher with FloCo than with monopolar encoding. In the right liver lobe, the effect size was less, with approximately 10% to 15% increased SNR with FloCo than with monopolar encoding. The quantitative difference between the monopolar and FloCo ADCs was small, but the mixed ADC (calculated with b50-Monopolar and b800-FloCo) was reduced by approximately 15%. The differences between the three ADCs (Monopolar, FloCo, and Mixed) were significant (for the left and right liver lobe). The post hoc evaluation revealed no significant differences between monopolar and FloCo ADCs (in left and right liver lobes), but a significant difference was found between FloCo and mixed ADC (for left and right liver lobes).

**Table 3:** Descriptive statistics of the quantitative lesion evaluation.

	Liver lobe	Diffusion encoding	Mean ± Standard deviation	Minimum	Median [Q1 Q3]	Maximum	p-value (Shapiro–Wilk)	p-value (Mp vs FC)	p-value (post hoc test) (ADC: Mp vs FC, Mp vs mixed, FC vs mixed)
Lesion SNR (b800)	Left	Monopolar	15.24 ± 6.84	3.56	13.83 [10.17 20.01]	33.07	0.61	**0.007**	
		FloCo	20.20 ± 7.47	6.22	20.17 [16.56 25.85]	37.16	0.67		
	Right	Monopolar	19.62 ± 7.96	7.13	19.60 [13.32 24.12]	48.37	**< 0.01**	**< 0.001**	
		FloCo	23.46 ± 9.02	9.74	23.19 [16.68 28.39]	47.26	**0.047**		
Lesion ADC (μm²/ms)	Left	Monopolar	1.52 ± 0.61	0.81	1.34 [1.08 1.69]	3.53	**< 0.001**	**0.0175**	Mp/FC: 0.97 Mp/mixed: 0**.028** FC/mixed: 0**.0499**
		FloCo	1.45 ± 0.51	0.85	1.33 [1.14 .47]	3.27	**< 0.001**		
		Mixed	1.24 ± 0.45	0.68	1.12 [0.96 1.33]	2.81	**< 0.001**		
	Right	Monopolar	1.16 ± 0.40	0.21	1.19 [0.92 1.34]	2.56	**0.02**	**0.0159**	Mp/FC: 0.87 Mp/mixed: 0.071 FC/mixed: 0**.019**
		FloCo	1.20 ± 0.31	0.71	1.13 [1.00 1.34]	2.48	**< 0.001**		
		Mixed	1.03 ± 0.35	0.34	0.96 [0.84 1.09]	2.41	**< 0.001**		
Diameter (smallest lesion; mm)	Left	FloCo	6.99 ± 3.87	3.88	5.86 [5.05 6.99]	20.37			
	Right		8.15 ± 7.31	3.72	5.68 [4.94 7.57]	40.02			
Diameter (largest lesion; mm)	Left		32.47 ± 24.19	4.65	25.01 [15.22 42.70]	102.65			
	Right		35.54 ± 29.38	3.95	26.12 [13.48 52.63]	118.78			

Significant p-values are in bold font. Mixed ADC was calculated with b50 monopolar and b800 FloCo data. The parametric test was the Wilcoxon signed-rank test for the SNR and the Kruskal–Wallis test for the ADC. Q1 = Quartile 1, Q3 = Quartile 3. FC = FloCo = Flow-compensated. Mp = monopolar.

S1 Fig shows ADC maps computed with monopolar b50 data combined once with FloCo b800 data (i.e., mixed) and once with monopolar b800 data (i.e., monopolar) as well as ADC maps computed from FloCo data alone (i.e., FloCo). The mixed and monopolar maps are of similar quality, but the quality of the FloCo map is somewhat lower. This indicates that the proposed mixed-acquisition protocol (i.e., acquiring b50 monopolar and b800 FloCo data) may also yield ADC maps of sufficient quality.

An extended evaluation of the ADC maps was not performed because many ADC maps were corrupted by breathing motion (the influence of which is visible at the thick arrow). The use of navigator triggering might have mitigated this breathing-related problem but was not possible with the prototype sequence.

### Qualitative image analysis

**Fig 7:**
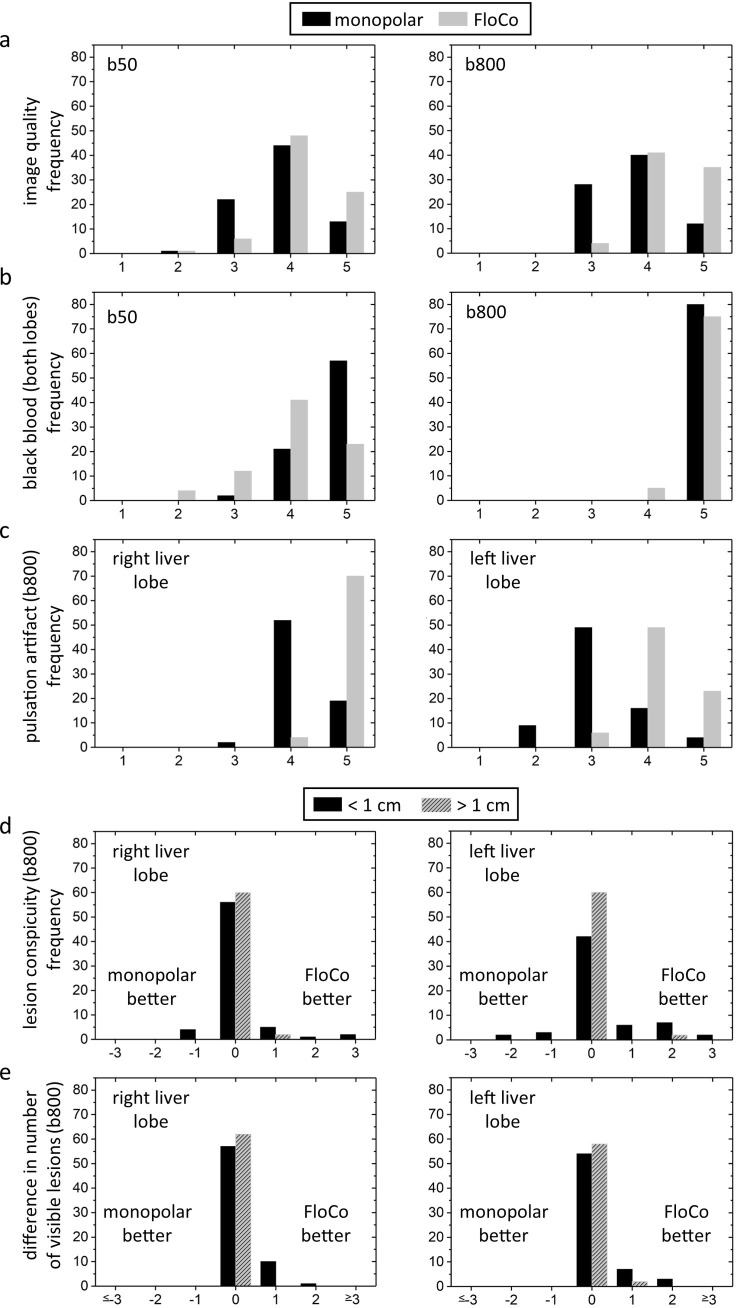
Histograms of the qualitative lesion evaluation. The scales in a) to d) range from 1 (worst) to 5 (best). Difference in number of visible lesions (Δ_lesions_) is plotted in e) and calculated as number of lesions visible with flow-compensated (FloCo) minus number of lesions visible with monopolar diffusion encoding. A positive score indicates that more lesions were found with FloCo diffusion encoding.

Fig 7 shows histograms of the Likert score evaluation for monopolar and FloCo diffusion encoding, with the pooled data of the two readers presented. Tables 4 and 5 show the individual reader data. The overall image quality was significantly better with the FloCo diffusion encoding at both b-values (for both readers). The black-blood state was reached significantly better at b50 with monopolar diffusion encoding (for both readers), but no significant difference was observed at b800 (p ≥ 0.25 for both readers). In contrast, the pulsation artifact was significantly less with FloCo diffusion encoding at both b-values (for both readers).

**Table 4:** Qualitative Likert score evaluation by reader 1 and reader 2 (same data as in Fig 7).

	Liver lobe	b-value	Diffusion encoding	Reader	1	2	3	4	5	p-value R1	p-value R2	κ
Image quality	Left & right	b50	Monopolar	R1	0	1	12	20	7	**0.008**	**0.001**	0.62
				R2	0	0	10	24	6			
			FloCo	R1	0	1	3	25	11			0.49
				R2	0	0	3	23	14			
	Left & right	b800	Monopolar	R1	0	0	14	19	7	**< 0.001**	**< 0.001**	0.51
				R2	0	0	14	21	5			
			FloCo	R1	0	0	3	19	18			0.68
				R2	0	0	1	22	17			
Black-blood signal	Left & right	b50	Monopolar	R1	0	0	2	9	29	**< 0.001**	**< 0.001**	0.82
				R2	0	0	0	12	28			
			FloCo	R1	0	3	6	19	12			0.72
				R2	0	1	6	22	11			
	Left & right	b800	Monopolar	R1	0	0	0	0	40	.50	.25	1.00
				R2	0	0	0	0	40			
			FloCo	R1	0	0	0	2	38			0.79
				R2	0	0	0	3	37			
Pulsation artifact	Left	b800	Monopolar	R1	0	5	25	7	2	**< 0.001**	**< 0.001**	0.67
				R2	0	4	24	9	2			
			FloCo	R1	0	0	4	24	11			0.75
				R2	0	0	2	25	12			
	Right	b800	Monopolar	R1	0	0	1	28	8	**< 0.001**	**< 0.001**	0.81
				R2	0	0	1	25	11			
			FloCo	R1	0	0	0	2	35			1.00
				R2	0	0	0	2	35			

Significant p-values are printed in bold font.

**Table 5:** Lesion conspicuity and the difference in detectable lesions (Δ_lesions_) (same data as in Fig 7).

	Liver lobe	b-value	Diffusion encoding	Lesion size	Reader	-3	-2	-1	0	1	2	3	p-value	κ
Lesion conspicuity	Left	b800	FloCo vs monopolar	Small	R1	0	1	1	22	3	4	1	0.078	0.81
					R2	0	1	2	22	3	3	1	0.197	
			FloCo vs monopolar	Large	R1	0	0	0	29	0	1	0	1.000	1.00
					R2	0	0	0	29	0	1	0	1.000	
	Right	b800	FloCo vs monopolar	Small	R1	0	0	2	28	2	1	1	0.375	0.91
					R2	0	0	2	28	3	0	1	0.531	
			FloCo vs monopolar	Large	R1	0	0	0	30	1	0	0	1.000	1.00
					R2	0	0	0	30	1	0	0	1.000	
Δ_lesions_	Left	b800	FloCo vs monopolar	Small	R1	0	0	0	27	4	1	0	0.063	0.89
					R2	0	0	0	27	3	2	0	0.063	
			FloCo vs monopolar	Large	R1	0	0	0	29	1	0	0	1.000	1.00
					R2	0	0	0	29	1	0	0	1.000	
	Right	b800	FloCo vs monopolar	Small	R1	0	0	0	29	5	0	0	0.063	0.79
					R2	0	0	0	28	5	1	0	**0.031**	
			FloCo vs monopolar	Large	R1	0	0	0	31	0	0	0	1.000	1.00
					R2	0	0	0	31	0	0	0	1.000	

Significant p-values are printed in bold font. Lesion conspicuity: scores larger than zero indicate lesions that were better visible with FloCo encoding. Δ_lesions_: values larger than zero indicate that more lesions were detectable with FloCo encoding.

No negative values were calculated for Δ_lesions_, indicating that more or equal numbers of lesions were detected with FloCo diffusion encoding than with monopolar diffusion encoding for all participants. In the left liver lobe with FloCo diffusion encoding, readers 1 and 2 found six and seven additional small lesions, respectively, and one additional large FLL both. In the right liver lobe, readers 1 and 2 found five and seven additional small lesions, respectively, with FloCo diffusion encoding, and no additional large lesions.

Cohen’s κ was ≥ 0.67 in all considered cases, indicating *substantial* or *almost perfect* agreement according to Landis and Koch [35].

## Discussion

We evaluated the suitability of conventional monopolar and flow-compensated (FloCo) diffusion encoding for the detection of focal liver lesions in oncologic patients. With flow-compensated encoding, the pulsation artifact was reduced and more lesions were detected. Moreover, the overall image quality was superior and the blood was sufficiently dark at b800. Thus, flow-compensated encoding appears to be better suited for the detection of focal liver lesions.

Different techniques have been described to reduce pulsation artifacts in the liver. In this work we focused on a modified DWI sequence with FloCo diffusion encoding. Yet, there are alternative approaches, one of which is electrocardiogram (ECG) triggers [36, 37]. However, several drawbacks of ECG triggering impede its widespread use in clinical DWI. It is cumbersome to use the ECG trigger, and the rapid switching of diffusion gradient often degrades the ECG signal quality. Breathing navigator triggers have also been recommended, particularly for the detection of small FLLs [31]. However, using ECG and breathing triggers simultaneously would presumably extend the scan time to an unacceptable degree.

The pulsation artifact can also be reduced with postprocessing techniques. Ichikawa et al. and Liau et al. proposed two approaches involving averaging multiple acquired images such that stronger signal intensities are more heavily weighted [38, 39]. This reduces the signal void, a characteristic of the pulsation artifact, in the averaged images. Similar methods could be applied to FloCo diffusion encoding data, but a straightforward application might be difficult to achieve. For example, the blood signal was sufficiently dark at b800 in the averaged images investigated in our study, but this is not necessarily true for each acquired FloCo image, unlike for the monopolar images [25]. The described weighted average approaches could then lead to a bright blood signal at some image positions. Thus, we did not use advanced postprocessing approaches in our current study, although we deem it likely that they could lead to further improvements if carefully adapted.

At b50, the black-blood property received mostly high ratings (i.e., Likert scores of 4 and 5, c.f. Fig 7) for both diffusion encoding methods, though monopolar generally performed better. At b800, this difference virtually disappeared. This is consistent with Rauh et al.’s [25] proposal to use monopolar diffusion encoding for b50 images and FloCo diffusion encoding for b800 images. ADC maps might then be computed from b50-Monopolar and b800-FloCo data (S1 Fig). In our setup, images acquired with two consecutively run sequences were combined for this purpose. As readjustments between the sequences may potentially alter image intensities, a more favorable approach in future implementations would be to acquire all data within one sequence thus avoiding potentially occurring biases due to readjustments between the sequences. An alternative approach to suppress the blood signal at low b-values is to use partially flow-compensated diffusion encoding [26, 40].

The finding that the SNR was increased with FloCo diffusion encoding may be interpreted as a reduction of the pulsation artifact. But this interpretation is limited in so far as the signal decay due to the IVIM effect is also reduced for FloCo diffusion encoding. [18].

Concerning the ADC, the different temporal spectrum of the monopolar and FloCo diffusion encoding [11, 12, 41] might have some influence altering the ADC, although we believe that the quantitative impact on the ADC should be small, i.e. on the order of below 10%, given the similar slopes of the signal decay curves observed by Wetscherek et al. in the liver for monopolar and FloCo diffusion encoding [18]. The occurrence of the smaller ADC values observed for the mixed approach can be well explained by the IVIM model in the ballistic limit [17]. The monopolar signal at b50 experiences the IVIM-related signal drop, but the FloCo signal at b800 does not (in the ballistic limit). Hence, the signal drops less from b50 to b800 compared to the “not mixed” approach–and the ensuing ADC is decreased.

We used the minimal TE achievable with the FloCo sequence at b800 both for FloCo and monopolar diffusion encoding (i.e., TE = 70 ms, TE = 46 ms would have been possible with monopolar diffusion encoding). The TE of 70 ms lies within the values used in other patient studies, which range from 49.7 ms to 82 ms at 1.5 T [42–46]. Although Taouli and Koh [47] suggested in their review to use the minimal achievable TE, which they found to be approximately 71 ms with their system, it seems reasonable to maximize the CNR. Assuming that the proton density and the noise are identical in liver and lesion, the respective optimal TE is achieved when the contrast between liver and lesion becomes maximal, i.e. at

$${\text{TE}}_{\text{opt}}=\frac{1}{T_{2,\text{liver}}^{-1}-T_{2,\text{lesion}}^{-1}}\log\frac{T_{2,\text{lesion}}}{T_{2,\text{liver}}},$$

with the transversal relaxation times *T*_2_,_liver_ and *T*_2_,_lesion_ of liver tissue and lesion, respectively. For example, Cieszanowski et al. reported *T*_2_,_liver_ = 54 ms and *T*_2_,_lesion_ = 85 ms at 1.5 T. Consequently, TEopt ≈ 67 ms, which is very close to the setting that we had used (TE = 70 ms). Thus, the fact that the minimally achievable echo time is prolonged with the FloCo diffusion encoding is presumably not a decisive disadvantage in clinical practice.

The difference in found lesions, Δ_lesions,_ was rated significantly different between the two acquisition schemes by reader 1 for small lesions in the right liver lobe (but not by reader 2). Both readers did not find a significant difference for the left liver lobe. This is somewhat astonishing, since the left liver lobe is more prone to the pulsation artifact, but may be explained by the smaller size of the left liver lobe and the therewith reduced total number of lesions. The p-value was indeed only slightly above the significance threshold (p = 0.063) for the left liver lobe. Thus, although not significant, the probability that this result occurred by chance is only 6.3% (with the null hypothesis that no effect was present).

This study has several limitations. First, the acquisition was performed in the free-breathing mode because the prototype sequence did not allow for breathing navigator triggering. Particularly for smaller lesions, the use of breathing navigator triggering would presumably have been advantageous [31]. However, both DWI sequences were scanned in free breathing allowing for a fair comparison. Second, the TR was relatively long due to FloCo diffusion encoding posing high demands on the gradient system cooling rate. This drawback will likely become less severe as new generations of high-performance gradient systems are developed. Third, only one scanner from one vendor at one site was used in this study, potentially reducing the generalizability of the obtained results. Yet, we used a widespread 1.5 T MRI scanner from a large vendor. Finally, the malignancy of the lesions was not confirmed histologically for all lesions, causing us to rely on radiological classification. However, the read was performed by board-certified radiologists with extensive clinical experience in classification of liver lesions. The remaining uncertainty on the true lesion status should not be a major limitation of our study, which was focused on lesion conspicuity rather than on differentiating lesion types.

## Conclusions

In conclusion, the use of liver diffusion-weighted imaging with flow-compensated diffusion encoding is feasible on a currently widespread clinical 1.5 T MR scanner. The previously observed reduction in pulsation artifacts with flow-compensated diffusion encoding in healthy young volunteers was reproduced in older patients with focal liver lesions. We found that artifact reduction supported the detection of focal liver lesions compared to the conventional monopolar diffusion encoding by increasing the number of visible lesions.

## Supporting information

S1 Fig ADC maps computed with monopolar b50 data combined once with FloCo b800 data (i.e., mixed) and once with monopolar b800 data (i.e., monopolar) as well as ADC maps computed from FloCo data alone (i.e., FloCo). (TIF)

S1 Table Minimal data set of this study. (XLSX)

## Abbreviations

ADC: Apparent diffusion coefficient
DWI: diffusion-weighted imaging
EPI: echo planar imaging
FLL: focal liver lesion
FloCo: flow-compensated
GRAPPA: GeneRalized Autocalibrating Partial Parallel Acquisition
IVIM: intravoxel incoherent motion
RECIST: Response Evaluation Criteria in Solid Tumors
SNR: signal-to-noise ratio
TE: echo time
TR: repetition time
