# Supplementary figures and images for: Flow-compensated diffusion encoding in MRI for improved liver metastasis detection
Source: PLoS One. 2022 May 26;17(5):e0268843. doi: 10.1371/journal.pone.0268843 (PMC9135229; doi:10.1371/journal.pone.0268843)

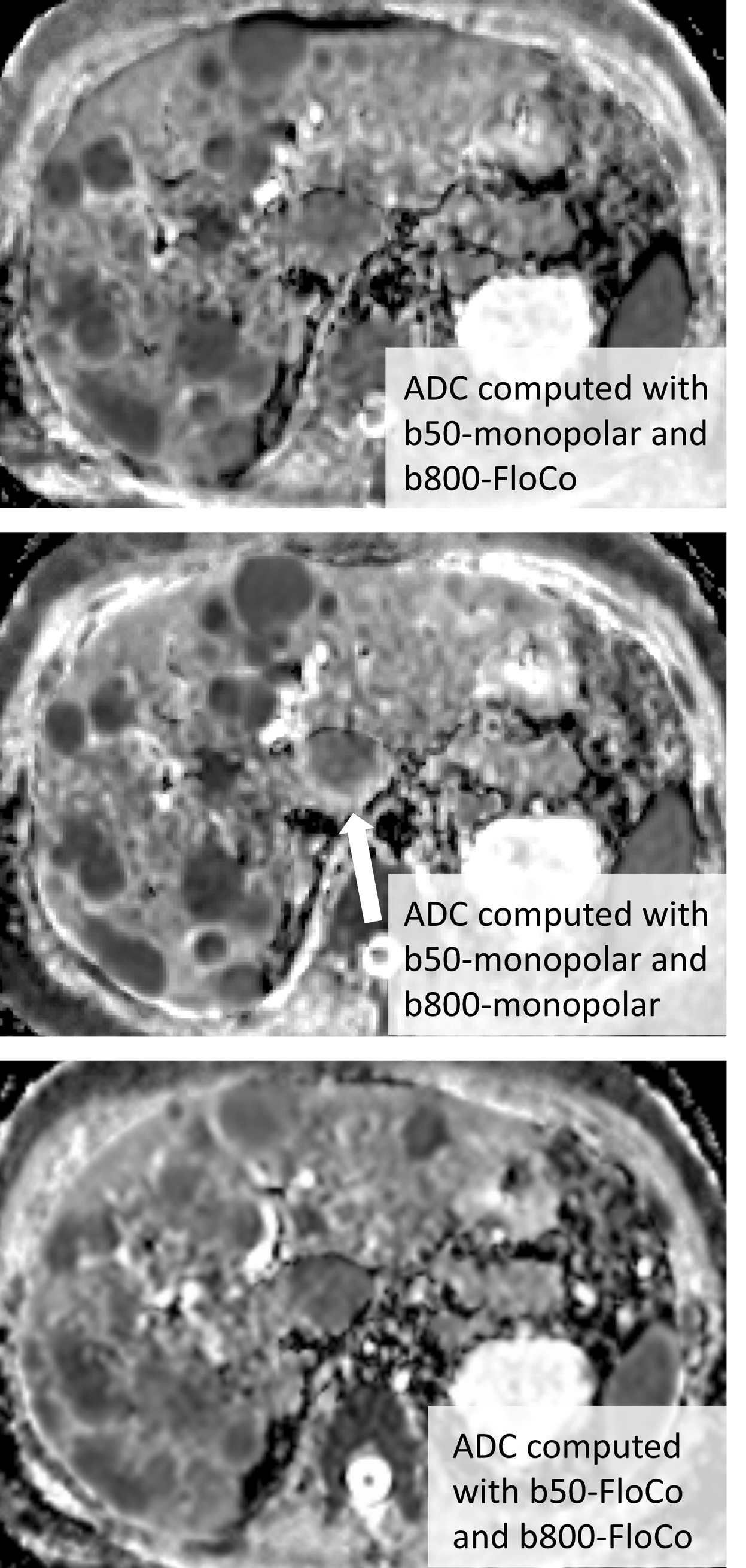

Supplement: S1 Fig — (TIF) [file pone.0268843.s001.tif]
